# Prediction Model for Early-Stage Pancreatic Cancer Using Routinely Measured Blood Biomarkers

**DOI:** 10.1001/jamanetworkopen.2023.31197

**Published:** 2023-08-28

**Authors:** Lenka N. C. Boyd, Mahsoem Ali, Annalisa Comandatore, Ingrid Garajova, Laura Kam, Jisce R. Puik, Stephanie M. Fraga Rodrigues, Laura L. Meijer, Tessa Y. S. Le Large, Marc G. Besselink, Luca Morelli, Adam Frampton, Hanneke W. M. van Laarhoven, Elisa Giovannetti, Geert Kazemier

**Affiliations:** 1 Department of Surgery, Amsterdam University Medical Center (UMC), Vrije Universiteit, Amsterdam, the Netherlands; 2 Laboratory of Medical Oncology, Department of Medical Oncology, Amsterdam UMC, Vrije Universiteit, Amsterdam, the Netherlands; 3 Imaging and Biomarkers, Cancer Center Amsterdam, Amsterdam, the Netherlands; 4 General Surgery Unit, Department of Translational Research and New Technologies in Medicine and Surgery, University of Pisa, Pisa, Italy; 5 Medical Oncology Unit, University Hospital of Parma, Parma, Italy; 6 Department of Surgery, Amsterdam UMC, University of Amsterdam, Amsterdam, the Netherlands; 7 Department of Surgery and Cancer, Hammersmith Hospital, Imperial College London, London, United Kingdom; 8 Department of Medical Oncology, Amsterdam UMC, University of Amsterdam, Amsterdam, the Netherlands; 9 Cancer Pharmacology Laboratory, Associazione Italiana per la Ricerca sul Cancro (Italian Association for Cancer Research) Start-Up Unit, Fondazione Pisana per la Scienza, Pisa, Italy

## Abstract

**Importance:**

Accurate risk prediction models using routinely measured biomarkers—eg, carbohydrate antigen 19-9 (CA19-9) and bilirubin serum levels—for pancreatic cancer could facilitate early detection of pancreatic cancer and prevent potentially unnecessary diagnostic tests for patients at low risk. An externally validated model using CA19-9 and bilirubin serum levels in a larger cohort of patients with pancreatic cancer or benign periampullary diseases is needed.

**Objective:**

To assess the discrimination, calibration, and clinical utility of a prediction model using readily available blood biomarkers (carbohydrate antigen 19-9 [CA19-9] and bilirubin) to distinguish early-stage pancreatic cancer from benign periampullary diseases.

**Design, Setting, and Participants:**

This diagnostic study used data from 4 academic hospitals in Italy, the Netherlands, and the UK on adult patients with pancreatic cancer or benign periampullary disease treated from 2014 to 2022. Analyses were conducted from September 2022 to February 2023.

**Exposures:**

Serum levels of CA19-9 and bilirubin from samples collected at diagnosis and before start of any medical intervention.

**Main Outcomes and Measures:**

Discrimination (measured by the area under the curve [AUC]), calibration, and clinical utility of the prediction model and the biomarkers, separately.

**Results:**

The study sample comprised 249 patients in the development cohort (mean [SD] age at diagnosis, 67 [11] years; 112 [45%] female individuals), and 296 patients in the validation cohort (mean [SD] age at diagnosis, 68 [12] years; 157 [53%] female individuals). At external validation, the prediction model showed an AUC of 0.89 (95% CI, 0.84-0.93) for early-stage pancreatic cancer vs benign periampullary diseases, and outperformed CA19-9 (difference in AUC [ΔAUC], 0.10; 95% CI, 0.06-0.14; *P* < .001) and bilirubin (∆AUC, 0.07; 95% CI, 0.02-0.12; *P* = .004). In the subset of patients without elevated tumor marker levels (CA19-9 <37 U/mL), the model showed an AUC of 0.84 (95% CI, 0.77-0.92). At a risk threshold of 30%, decision curve analysis indicated that performing biopsies based on the prediction model was equivalent to reducing the biopsy procedure rate by 6% (95% CI, 1%-11%), without missing early-stage pancreatic cancer in patients.

**Conclusions and Relevance:**

In this diagnostic study of patients with pancreatic cancer or benign periampullary diseases, an easily applicable risk score showed high accuracy for distinguishing early-stage pancreatic cancer from benign periampullary diseases. This model could be used to assess the added diagnostic and clinical value of novel biomarkers and prevent potentially unnecessary invasive diagnostic procedures for patients at low risk.

## Key Points

**Question:**

Can routinely measured blood biomarkers be used to develop and externally validate a prediction model that reliably detects early-stage pancreatic cancer?

**Findings:**

This diagnostic study including 545 adult patients with pancreatic cancer or benign periampullary disease found that a prediction model using commonly measured blood markers showed excellent discriminative performance and could be used to reduce the number of unnecessary biopsy procedures by 6% without missing early-stage pancreatic cancer in patients.

**Meaning:**

The findings of this diagnostic study suggest that in patients with suspected pancreatic cancer, this simple prediction rule may improve detection of early-stage pancreatic cancer.

## Teaser

This multicenter diagnostic study assessed a prediction model that uses readily available blood biomarkers to possibly distinguish early-stage pancreatic cancer from benign periampullary diseases.

## Introduction

A combination of clinical symptoms, carbohydrate antigen 19-9 (CA19-9) serum levels, radiological findings, and pathologic confirmation of the diagnosis by fine-needle aspiration or brush cytology are the current modalities used for diagnosing pancreatic ductal adenocarcinoma (PDAC).^1^ This diagnostic process is time consuming and the diagnosis often remains inconclusive until after an invasive biopsy or resection of the pancreatic tumor has been performed. Therefore, it would be desirable to investigate a more efficient, accurate, and minimally invasive method for diagnosing PDAC, one that reduces time to treatment and prevents unnecessary diagnostic measures.

 With this goal in mind, numerous research groups have recently tried to identify PDAC-specific blood-based biomarkers, including circulating tumor cells, cell-free tumor DNA, exosomes, and serum proteins.^2,3,4^ However, none of these models have entered clinical practice yet. Ideally, risk scores using routinely measured biomarkers, such as CA19-9 and bilirubin levels, could be used to detect early-stage pancreatic cancer, and prevent potentially unnecessary and invasive diagnostic procedures for patients with benign pancreatic diseases that are suggestive of pancreatic cancer.^5^ These models could additionally be used to screen novel tumor biomarkers for their added value and identify potentially promising markers with sufficient incremental diagnostic and clinical utility to warrant further validation.^5,6^

Synthesized by normal human pancreatic and biliary ductal cells, CA19-9 in small amounts are commonly detectable in human serum. Elevated levels of CA19-9 are associated with PDAC or distal cholangiocarcinoma and may also be found in benign gastrointestinal disorders often characterized by obstructive jaundice.^1^ Bilirubin originates from hemoglobin of senescent erythrocytes, which accounts for approximately 80% of bilirubin in the human body. Levels of bilirubin can be elevated when there is an obstruction of the biliary tree, either due to a benign disorder or a malignant neoplasm.^7^ In the case of malignant disease, bilirubin is known to reach higher levels. Although the precise dynamic between CA19-9 and bilirubin has not been clarified yet, literature suggests that the biliary tract functions as an excretion pathway for CA19-9 to the liver. Therefore, obstructions in this pathway may cause hyperbilirubinemia, which may produce elevated levels of CA19-9, and thereby reduce the specificity of CA19-9 in diagnosing hepatopancreaticobiliary disease. Adjusting CA19-9 to bilirubin could improve the diagnostic value of CA19-9 for distinguishing between benign and malignant hepatopancreaticobiliary diseases.

Our recent meta-analysis^4^ demonstrated that the most researched novel blood-based proteins for PDAC had a lower diagnostic power than CA19-9 and did not have added diagnostic value in combination with CA19-9. In addition, the implemented study designs were not relevant to a realistic clinical setting, despite the widespread use of these designs, such as diagnostic case-control designs with patients with PDAC and healthy controls. These findings provide a clear suggestion that research focusing on the improvement of the diagnostic process for PDAC should commit to practicality, effectiveness, and reliability, thereby taking the clinical setting into account and exploiting the full potential of current practice and measurements. From this standpoint, further research on tumor-specific biomarkers could be facilitated by constructing a solid basis of a model and workflow to which newly found markers can be added—concomitantly reducing waste of laboratorial costs and efforts.

These principles formed the core for analysis in an initial study population,^5^ in which internal validation indicated an added value of this model including the CA19-9:bilirubin ratio. In the current study, we redeveloped our model with a larger development cohort, and we externally validated it. Additionally, we assessed its discriminative performance, calibration, and clinical utility in a population of patients with early-stage PDAC and benign periampullary diseases.

## Methods

The study design and protocol were approved by the Medical Ethics Board of Amsterdam UMC. Informed consent was obtained both orally and in written form. We followed the Transparent Reporting of a Multivariable Prediction Model for Individual Prognosis or Diagnosis (TRIPOD) reporting guidelines, as well as the TRIPOD for Abstracts guidelines (eMethods in Supplement 1).^8^

### Study Design

Patients were recruited from 4 participating centers: Amsterdam UMC (the Netherlands), University of Pisa (Italy), University of Parma (Italy), and Imperial College London (UK). Patients who visited the hepatopancreaticobiliary clinic in 2015 through 2020 were included, and their clinicopathological characteristics (eg, sex, serum CA19-9 and bilirubin levels, final diagnosis) were collected in a prospectively maintained database. Patients with PDAC and benign periampullary diseases were included, and the final diagnosis was based on histopathologic confirmation of the biopsy or resection specimen. Patients with benign periampullary diseases had the following final diagnoses: benign central bile duct stenosis, cholangitis, chronic pancreatitis, intraductal papillary mucinous neoplasm, pancreatic cysts, or pancreatic lipomas. Patients with primary cholecystitis, hepatitis, liver cirrhosis, neuroendocrine tumors, and sclerosing cholangitis were excluded.^5^

The CA19-9 expression levels were determined by the Immunometric assay, Luminescence (Advia Centaur XP, Siemens Healthineers) and bilirubin levels by the colorimetric diazo method (Bilirubin Total Generation 3, Roche International, Rotkreuz, Switzerland). Serum levels of CA19-9 and bilirubin were measured at diagnosis, before the start of any therapy.

### Statistical Analysis

Continuous variables were reported as mean (SDs) or as median (IQR), and were compared using Student t test or the Mann-Whitney U test, as appropriate. Categorical variables were summarized as frequencies and percentages and were compared using χ^2^ tests. There were no missing data in the predictor variables (ie, CA19-9 and bilirubin) used in the model.

Differences in patient characteristics between the development and validation cohort were assessed with membership models; a C statistic of the membership model close to 0.5 indicated no substantial differences in patient characteristics between the development and validation cohort; a membership model C statistic close to 1 indicated the opposite.^9,10^

A previously developed model^5^ (using CA19-9 and bilirubin levels and the CA19-9:bilirubin ratio) was redeveloped in an expanded cohort (ie, the original development cohort of patients from Amsterdam UMC combined with patients from Imperial College London). The model was externally validated in a validation cohort (University of Pisa and University of Parma) in accordance with minimum sample size requirements for clinical prediction models.^5,11,12^ Sample size calculations are provided in the eMethods in Supplement 1. Briefly, the sample size of the current study was sufficient to develop a clinical prediction model using 9 predictor parameters (including nonlinear terms) and to validate the model.

In accordance with the TRIPOD statement, the performance of the prediction model was assessed in terms of discrimination, calibration, and clinical utility. The discrimination of the model (ie, the degree to which the model can differentiate between patients with early-stage PDAC and with benign diseases) was evaluated using ROC (receiver operating characteristic) curves and the area under the ROC curve (AUC). Briefly, the AUC ranges from 0.5 (no better than chance) to 1.0 (perfect discrimination) with higher AUC values indicating a higher discriminative performance of the test.^10^

Calibration refers to the degree of agreement between predicted and observed risks and was assessed using visual inspection of flexible calibration curves, a recalibration test, and the integrated calibration index (ICI). A calibration curve graphically shows the association between the predicted and the observed probability of a certain disease or outcome. Good calibration is present if the calibration curve closely follows the diagonal identity line of perfect calibration, represented by a line with an intercept of 0 and a slope of 1. The recalibration test is a likelihood ratio test for the null hypothesis that the calibration intercept is 0 and the slope is 1. A *P* value higher than .05 for the recalibration test indicated the absence of evidence that the model is systematically miscalibrated, whereas the calibration curve allows both systematic and local miscalibration patterns to be detected (eg, overestimated risks for low-risk patients and underestimated risks for high-risk patients). The ICI is the weighted difference between predicted and observed risks, ie, values closer to 0 indicate better calibration.

The clinical utility of the model was assessed using decision curve analysis. Briefly, the clinical utility of a prediction model is evaluated in a decision curve analysis by calculating the standardized net benefit (sNB) over a range of decision thresholds and by comparing the sNB of the prediction model to other diagnostic strategies, including a *test all* and a *test none* approach. A model with the highest sNB across all clinically plausible thresholds represents the best diagnostic strategy among all alternatives. In a decision curve analysis, the threshold represents the minimum probability of disease before certain interventions, such as a biopsy procedure, are considered. Thus, low decision thresholds can be considered for situations in which finding a true-positive result is deemed to be more important than an unnecessary test, ie, a false-positive.

Statistical tests were 2-sided and a *P* value < .05 was considered to be statistically significant. There were no missing data in model variables. All analyses were performed in R, version 4.2.1 (R Foundation for Statistical Computing), and Stata, version 17.0 (StataCorp LLC).

## Results

### Study Population

**Figure 1.**
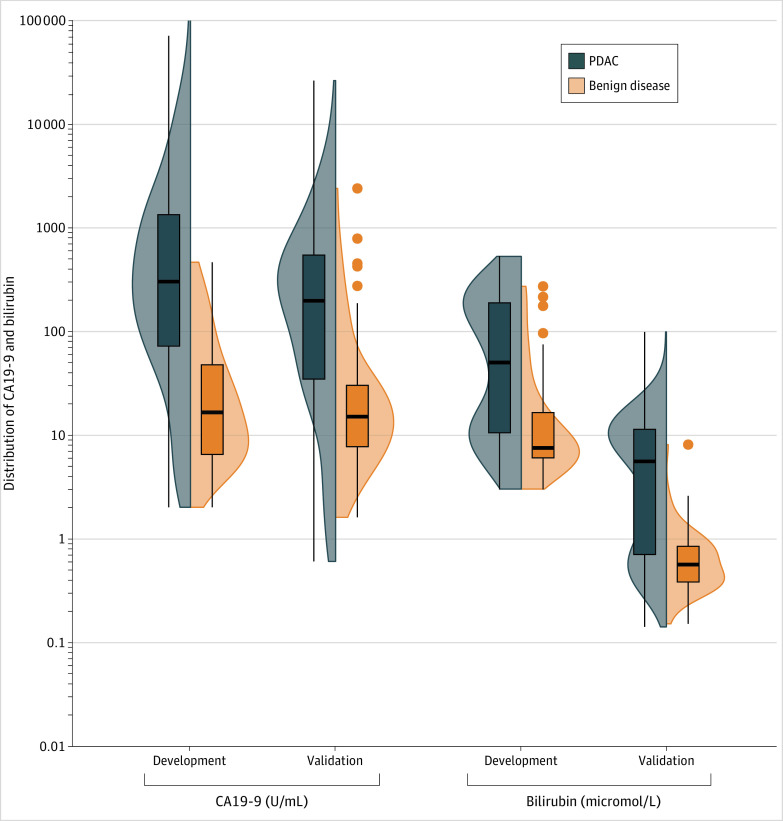
Distribution of CA19-9 and Bilirubin Serum Levels in the Development and Validation Cohorts Side-by-side box-and-violin plot of CA19-9 and bilirubin serum levels in the development and validation cohort for PDAC (blue) and benign disease (orange). CA19-9 refers to carbohydrate antigen 19-9 and PDAC, to pancreatic ductal adenocarcinoma.

The study sample included 249 patients in the development data set (mean [SD] age at diagnosis, 67 [11] years; 112 [45% female and 137 [55%] male individuals) and 296 patients in the validation data set (mean [SD] age at diagnosis, 68 [12] years; 157 [53%] female and 139 [47%] male individuals) (Table). Race and ethnicity data were not routinely collected by these hospitals and therefore, were not considered. The validation set differed considerably from the development set in terms of patient characteristics (membership model C statistic, 0.92; 95% CI, 0.90-0.94). Specifically, and as shown in Figure 1, bilirubin values of patients with PDAC were considerably lower in the validation cohort (median [IQR], 6 μmol/L [1-11] vs 53 μmol/L [10-191]; *P* < .001). Lower CA19-9 values were also observed for patients with PDAC in the validation cohort compared with the development cohort (median [IQR]; 197 U/mL [35-545] vs 326 U/mL [76-1503]; *P* = .002), as well as lower bilirubin values for patients with benign disease in the validation cohort compared with the development cohort (median [IQR], 0.6 μmol/L [0.4-0.8] vs 8 μmol/L [6-15]; *P* < .001).

**Table.** Baseline Characteristics of the Included Patients With PDAC or Benign Disease

Characteristic	Development cohort (Amsterdam UMC and Imperial College)		Validation cohort (University of Pisa and University of Parma)	
	PDAC (n = 182)	Benign disease (n = 67)	PDAC (n = 221)	Benign disease (n = 75)
Age, mean (SD), y	69 (10)	65 (15)	70 (11)	64 (13)
Sex, No. (%)				
Female	86 (47)	27 (40)	109 (49)	48 (64)
Male	96 (53)	40 (60)	112 (51)	27 (36)
Tumor stage, No. (%)^a^				
Stage I-II	79 (43)	0	221 (100)	0
Stage III-IV	91 (50)	0	0	0
NA	12 (7)	67 (100)	0	75 (100)
CA19-9, median (IQR), U/mL	326 (76-1503)	18 (6-47)	197 (35-545)	14 (7-30)
CA19-9, No. (%)^c^				
Normal	28 (15)	41 (69)	56 (25)	59 (79)
Elevated	154 (85)	18 (31)	165 (75)	16 (21)
Bilirubin, median (IQR), μmol/L	53.0 (10.0-191.0)	8.0 (6.0-15.0)	6.0 (1.0-11.0)	0.6 (0.4-0.8)
Bilirubin, No. (%)^b^				
Normal	73 (40)	48 (83)	211 (95)	75 (100)
Elevated	109 (60)	10 (17)	10 (5)	0

Abbreviations: CA19-9, carbohydrate antigen 19-9; dCCA, distal cholangiocarcinoma; NA, not applicable; PDAC, pancreatic ductal adenocarcinoma; UMC, University Medical Center.

^a^ Per the *American Joint Committee Cancer Staging Manual*, 8th ed.

^b^ Serum levels of CA19-9 >37 U/mL and bilirubin >20 μmol/L were considered to be elevated.

### Performance of the Prediction Model

**Figure 2.**
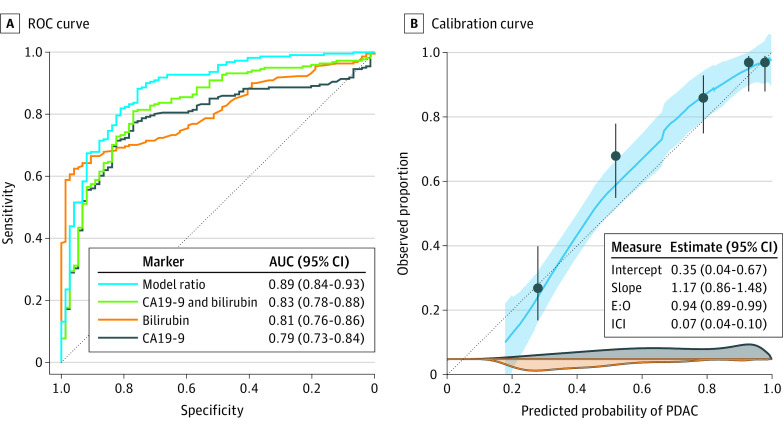
Discrimination and Calibration of the Prediction Model for Early-Stage PDAC vs Benign Disease A, Receiver operating characteristic (ROC) curves for the risk prediction model, CA19-9, bilirubin, and both CA19-9 and bilirubin. B, LOESS-smoothed flexible calibration curve (light blue) with 95% CIs (shaded area), grouped proportions (dots) with 95% CIs, and kernel density estimates of the predicted risks for patients with PDAC (dark blue density plot), and patients with benign disease (orange density plot). The gray oblique reference line indicates perfect calibration with an intercept of 0, a slope of 1, an E:O ratio of 1, and an ICI of 0. AUC refers to area under the receiver operating characteristic curve; CA19-9, carbohydrate antigen 19-9; E:O, ratio of expected to observed number of patients with PDAC; ICI, integrated calibration index; LOESS, locally estimated scatterplot smoothing; PDAC, pancreatic ductal adenocarcinoma; and ROC, receiver operating characteristic.

As show in Figure 2, at external validation the model demonstrated high discriminative performance with an area under the ROC curve (AUC) of 0.89 (95% CI, 0.84-0.93) and outperformed CA19-9 (ΔAUC, 0.10; 95% CI, 0.06-0.14; *P* < .001), bilirubin (ΔAUC, 0.07; 95% CI, 0.02-0.12; *P* = .004), and a prediction model using both CA19-9 and bilirubin (ΔAUC, 0.05; 95% CI, 0.03-0.08; *P* = .0002). The model also showed high discriminative performance in the subset of patients without elevated CA19-9 levels (AUC, 0.84; 95% CI, 0.77-0.92). Although there was no evidence that the model was systematically miscalibrated (recalibration test, *P* = .26), the calibration intercept (0.35; 95% CI, 0.04-0.67) and calibration slope (1.17; 95% CI, 0.86-1.48) indicated that the model underestimated the risk of PDAC on average. Additionally, the calibration curve indicated that the prediction model overestimated the risk of early-stage PDAC for patients at low risk.

### Clinical Utility of the Prediction Model

**Figure 3.**
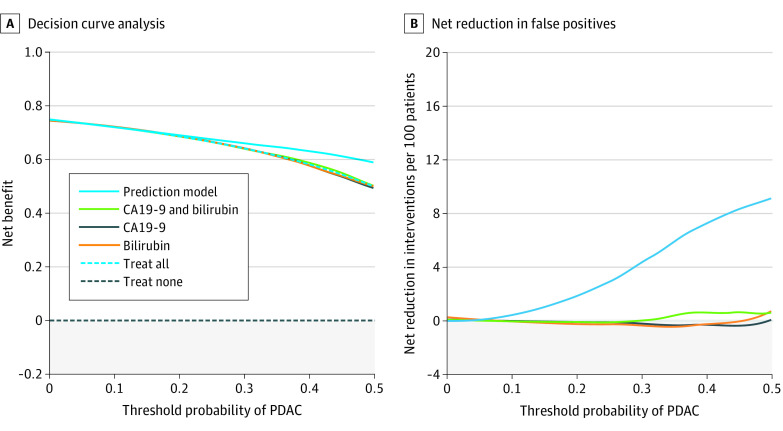
Clinical Utility of the Prediction Model for Early-Stage PDAC vs Benign Disease A, Decision curve analysis showing the net clinical benefit for different management strategies. B, Net reduction in unnecessary diagnostic interventions (ie, false-positive results) per 100 patients. PDAC refers to pancreatic ductal adenocarcinoma.

Decision curve analyses are shown in Figure 3 for a biopsy specimen based on the prediction model approach; a biopsy specimen based on bilirubin−CA19-9 (both) approach; a biopsy specimen for all patients approach; and a biopsy specimen for none approach. At each risk threshold, the clinical utility of the prediction model was either equal to or higher than all other approaches. At a risk threshold (ie, the minimum probability of PDAC before a biopsy is considered) greater than 6%, the clinical utility of a risk-score-based biopsy approach was the highest. For instance, at a risk threshold of 30% (the prevalence of PDAC in suspected pancreatic cancer^13^), decision curve analysis indicated that performing a biopsy procedure based on the prediction model was equivalent to reducing the biopsy rate by 6% (95% CI, 1%-11%), without missing early-stage PDAC cases (Figure 3).

## Discussion

In this diagnostic accuracy study, a simple prediction rule for early-stage PDAC was redeveloped in a larger cohort and externally validated. At external validation, our model demonstrated excellent discriminative performance (AUC 0.89), was adequately calibrated, and showed clinical utility at relevant decision thresholds. Decision curve analysis indicated that the prediction model could prevent unnecessary diagnostic testing in patients with a low risk for PDAC.

The primary strength of our model is that it relies exclusively on readily available laboratory tests, whereas previous predictive models for PDAC have traditionally focused on novel blood-based markers that are not routinely measured in the clinic, such as microRNAs, inflammation-associated proteins, and circulating tumor DNA.^4,14,15,16,17^ In addition, our model showed high discriminative performance in a population of early-stage PDAC patients and benign periampullary diseases. This is important because distinguishing PDAC from benign diseases is the primary diagnostic challenge.^18^ Most analyses are based on differentiating PDAC from healthy controls, which does not correspond to the clinical target population.^4^ In addition, diagnostic accuracy is considerably overestimated in studies using healthy controls compared with studies using a clinically representative sample.^19^

Ideally, prediction models for detecting PDAC should aim to distinguish early-stage PDAC from clinically similar benign diseases, and should demonstrate adequate discriminative performance, calibration, and clinical utility at external validation.^4^ Evaluating prediction models for their calibration and clinical utility, as determined through decision curve analysis, is of particular importance because the AUC of a prediction model is not directly related to a model’s potential clinical usefulness.^8^ A model with high diagnostic accuracy but poor calibration can negatively influence clinical decision-making, and can produce overtreatment and overdiagnosis if risk predictions are consistently too high, or undertreatment and underdiagnosis if risk predictions are consistently too low.^10^ These outcomes can also be assessed through decision curve analysis, which evaluates the potential clinical utility of risk scores, and is influenced by both diagnostic accuracy and calibration. In this study, our model was adequately calibrated and demonstrated clinical utility at relevant decision thresholds. However, further external validation is necessary to investigate the heterogeneity in the model’s performance, evaluate situations in which the model performs poorly, and recalibrate the model for specific clinical and geographic settings.

Notably, there were large differences in patient characteristics between the development and validation cohorts. Specifically, CA19-9 and bilirubin levels were considerably lower in the validation cohort, both for patients with PDAC and patients with benign periampullary diseases. This discrepancy was possibly caused by differences in patient selection. First, all patients with PDAC in the validation cohort had early-stage pancreatic cancer, whereas 50% of patients with PDAC in the development cohort had stages III to IV disease, which is associated with substantially higher bilirubin and tumor marker levels. Second, a higher percentage of patients with benign disease in the validation cohort were diagnosed with chronic pancreatitis, whereas relatively fewer patients were diagnosed with obstructive biliary diseases. This potentially explains the lower bilirubin values in the validation cohort, given that obstructive biliary diseases can elevate serum bilirubin values. Despite these differences in patient characteristics between the development and validation cohort, the model still performed well at external validation with high discriminative performance and adequate calibration, demonstrating the potential utility of this model in patient cohorts that do not closely resemble the situation of the development cohort. This is particularly of importance given that prediction models are known to show worse performance when externally validated in cohorts that differ in patient case mix (eg, if patients in the validation cohort have a lower tumor grade) and patient selection criteria.

Although novel biomarkers could potentially enhance diagnostic accuracy owing to their more tumor-specific nature, it is generally unclear what the incremental value of these markers would be beyond the simple laboratory tests and the readily available patient characteristics. Notably, a recent meta-analysis^4^ that used both aggregated and individual participant data showed that 6 frequently studied protein biomarkers did not improve diagnostic accuracy and clinical utility for detecting PDAC when combined with CA19-9 in a prediction model. As such, our model could potentially also be used to investigate which novel biomarkers show sufficient added value to warrant further investigation. This approach would allow potentially promising biomarkers to be screened for their incremental value and could facilitate an early identification of biomarkers that are unlikely to add meaningful diagnostic information to readily available tests.

### Limitations

The results of this diagnostic study should be assessed considering several limitations. Owing to sample size considerations, we did not incorporate other routinely measured laboratory variables, such as creatinine and hemoglobin, into our model.^17^ Additionally, we were not able to determine whether implementation of our model would improve clinical decision-making and patient outcomes in a prediagnostic setting of patients with suspected PDAC. These outcomes should be studied in diagnostic randomized clinical trials.

## Conclusion

At external validation, our prediction model using routinely measured blood markers showed high discriminative performance and adequate calibration. Decision curve analysis indicated that this model could be used to prevent unnecessary diagnostic testing in patients at low risk, without increasing the number of missed early-stage pancreatic cancer cases. Novel biomarkers should be assessed for their added diagnostic and clinical utility to our risk score in prospective single-gate cohort studies of patients with suspected pancreatic cancer.

## Notes

Supplement 1.
eMethods.

Supplement 2.
Data Sharing Statement
