# Supplementary material for: Prediction Model for Early-Stage Pancreatic Cancer Using Routinely Measured Blood Biomarkers
Source: JAMA Netw Open. 2023 Aug 28;6(8):e2331197. doi: 10.1001/jamanetworkopen.2023.31197 (PMC10463099; doi:10.1001/jamanetworkopen.2023.31197)
Supplement: Supplement 1. — eMethods. [file jamanetwopen-e2331197-s001.pdf]

# Supplemental Online Content

Boyd LNC, Ali M, Comandatore A, et al. Prediction model for early-stage pancreatic cancer using routinely measured blood biomarkers. *JAMA Netw Open.* 2023;6(8):e2331197. doi:10.1001/jamanetworkopen.2023.31197

**eMethods**

This supplemental material has been provided by the authors to give readers additional information about their work.

© 2023 Boyd LNC. *JAMA Netw Open.*

**eMethods.**

*Sample size calculation*

The sample size calculation for developing the model was based on the following assumptions: an overoptimism-corrected area under the receiver operating characteristic curve (AUC) of 0.90, a shrinkage factor of 0.90, a prevalence of PDAC in the development sample of 80%, and 9 model parameters (i.e., 3 model parameters per variable). As such, 246 patients (197 patients with pancreatic cancer and 49 patients with benign disease) were required.

The sample size for external validation of the model was based on closed-form solutions. A 95% CI width of 0.1 was targeted for both the observed:expected ratio and the AUC, assuming an O:E ratio of 1 and an AUC of 0.88 (i.e., an expected decrease in AUC of 0.02). This resulted in a minimum required sample size of 293 for the external validation set.

*Development and external validation of the model*

In the updated development set, a logistic regression model was developed using CA19-9, bilirubin, and an interaction term between CA19-9 and bilirubin. CA19-9 and bilirubin were modeled with nonlinear terms. All covariates were prespecified (i.e., predictor selection procedures were not used) to conserve the degrees of freedom spent in the modelling process.

*Performance measures of the model*

Discrimination was assessed using receiver operating characteristic (ROC) curves. The area under the ROC curve (AUC) was determined to assess the overall discrimination of the model. Confidence intervals of the AUC were calculated using the DeLong method, and bootstrapping was used to calculate the difference in AUC (with corresponding confidence intervals and *P*-values) between the prediction model and (1) CA19, (2) bilirubin, and (3) a logistic regression model with CA19-9 and bilirubin as predictors.

© 2023 Boyd LNC. *JAMA Netw Open.*

Calibration was assessed at four levels, in accordance with the calibration hierarchy proposed by Van Calster *et al*. First, at the lowest level (mean calibration), the expected-observed ratio was calculated with corresponding confidence intervals using bootstrap resampling. Second, weak calibration was assessed by calculating the intercept and slope of the model, and by using the Spiegelhalter *Z*-test, as well as a 2 *df* likelihood ratio test ('recalibration test') against the null hypothesis that the intercept is 0 and the slope is 1; thus, $P > .05$ in this test indicates absence of evidence that the model is systematically miscalibrated. Third, moderate calibration was assessed with flexible calibration curves using locally weighted scatterplot smoothing (LOESS), and with the integrated calibration index (ICI). Strong levels of calibration (i.e., the fourth and highest level of calibration) was evaluated by assessing model performance in the subset of patients with CA19-9 < 37 U/mL.

© 2023 Boyd LNC. *JAMA Netw Open.*
