# Supplementary material for: Prediction Model for Early-Stage Pancreatic Cancer Using Routinely Measured Blood Biomarkers
Source: JAMA Netw Open. 2023 Aug 28;6(8):e2331197. doi: 10.1001/jamanetworkopen.2023.31197 (PMC10463099; doi:10.1001/jamanetworkopen.2023.31197)
Supplement: Supplement 2. — Data Sharing Statement [file jamanetwopen-e2331197-s002.pdf]

Data Sharing Statement

Boyd. Prediction Model for Early-Stage Pancreatic Cancer Using Routinely Measured Blood Biomarkers. *JAMA Netw Open*. Published August 28, 2023. doi:10.1001/jamanetworkopen.2023.31197

Data
Data available: No

Additional Information
Explanation for why data not available: No informed consent from patients to share data in this way.
